# Impact of COVID-19 lockdown on the incidence and mortality of acute exacerbations of chronic obstructive pulmonary disease: national interrupted time series analyses for Scotland and Wales

**DOI:** 10.1186/s12916-021-02000-w

**Published:** 2021-05-17

**Authors:** Mohammad A. Alsallakh, Shanya Sivakumaran, Sharon Kennedy, Eleftheria Vasileiou, Ronan A. Lyons, Chris Robertson, Aziz Sheikh, Gwyneth A. Davies, on behalf of the EAVE II Collaborators, Colin R. Simpson, Jim McMenamin, Lewis D. Ritchie, Mark Woolhouse, Helen R. Stagg, Diogo Marques, Josie Murray, Sarah Stock, Rachael Wood, Colin McCowan, Utkarsh Agrawal, Annemarie B. Docherty, Rachel H. Mulholland, Emily Moore, James Marple, Vicky Hammersley

**Affiliations:** 1 Population Data Science, Swansea University Medical School, Data Science Building, Singleton Park, Swansea University, Swansea, SA2 8PP UK; 2 Health Data Research UK BREATHE Hub for Respiratory Health, University of Edinburgh, Edinburgh, EH8 9AG UK; 3 Health Protection Scotland, Public Health Scotland, Glasgow, UK; 4 Usher Institute, University of Edinburgh, Teviot Place, Edinburgh, EH8 9AG UK; 5 Department of Mathematics and Statistics, University of Strathclyde, Glasgow, UK

**Keywords:** Acute exacerbation of chronic obstructive pulmonary disease, COVID-19 lockdown

## Abstract

**Background:**

The COVID-19 pandemic and ensuing national lockdowns have dramatically changed the healthcare landscape. The pandemic’s impact on people with chronic obstructive pulmonary disease (COPD) remains poorly understood. We hypothesised that the UK-wide lockdown restrictions were associated with reductions in severe COPD exacerbations. We provide the first national level analyses of the impact of the COVID-19 pandemic and first lockdown on severe COPD exacerbations resulting in emergency hospital admissions and/or leading to death as well as those recorded in primary care or emergency departments.

**Methods:**

Using data from Public Health Scotland and the Secure Anonymised Information Linkage Databank in Wales, we accessed weekly counts of emergency hospital admissions and deaths due to COPD over the first 30 weeks of 2020 and compared these to the national averages over the preceding 5 years. For both Scotland and Wales, we undertook interrupted time-series analyses to model the impact of instigating lockdown on these outcomes. Using fixed-effect meta-analysis, we derived pooled estimates of the overall changes in trends across the two nations.

**Results:**

Lockdown was associated with 48% pooled reduction in emergency admissions for COPD in both countries (incidence rate ratio, IRR 0.52, 95% CI 0.46 to 0.58), relative to the 5-year averages. There was no statistically significant change in deaths due to COPD (pooled IRR 1.08, 95% CI 0.87 to 1.33). In Wales, lockdown was associated with 39% reduction in primary care consultations for acute exacerbation of COPD (IRR 0.61, 95% CI 0.52 to 0.71) and 46% reduction in COPD-related emergency department attendances (IRR 0.54, 95% CI 0.36 to 0.81).

**Conclusions:**

The UK-wide lockdown was associated with the most substantial reductions in COPD exacerbations ever seen across Scotland and Wales, with no corresponding increase in COPD deaths. This may have resulted from reduced transmission of respiratory infections, reduced exposure to outdoor air pollution and/or improved COPD self-management.

**Supplementary Information:**

The online version contains supplementary material available at 10.1186/s12916-021-02000-w.

## Background

The COVID-19 pandemic and subsequent challenges to healthcare systems have led to unprecedented disruptions of routine care for people with chronic conditions. In response to a surge in COVID-19 cases, the UK and devolved governments announced the first nationwide lockdown on 23rd March 2020, thereby severely restricting movements and social contacts [1]. The accompanying messages to avoid overwhelming the National Health Service (NHS) and fear of contracting SARS-CoV-2 in healthcare settings had an impact on people’s willingness to seek emergency care [2].

There is evidence that the UK-wide lockdown was associated with poorer cardiovascular [3] and cancer [4] outcomes, but its impact on serious chronic obstructive pulmonary disease (COPD) outcomes remains unclear.

Despite the disruption to routine COPD care [5, 6], the societal changes associated with lockdown—in particular, improvements in air quality and reductions in other viruses responsible for acute respiratory tract infections [7, 8]—may have led to an overall improvement in COPD outcomes. The available body of evidence suggests that there may have been a reduction in acute exacerbations of COPD (AECOPD), but these data are difficult to interpret because of methodological limitations including studying selective populations and/or from a limited number of centres [9–26]. Despite the changes in healthcare-seeking behaviour during the lockdown, people with severe AECOPD were still likely to seek medical attention as the symptoms are intense such that they are difficult to tolerate at home [12].

We sought to investigate the impact of the UK-wide COVID-19 lockdown on the overall numbers of recorded severe AECOPDs leading to admission and/or death across the entire populations of Scotland and Wales. To contextualise the findings, we also investigate AECOPDs that were recorded in primary care and emergency departments (EDs) in Wales.

## Methods

### Data sources, populations and case definitions

The study was based on the entire populations of Scotland and Wales (the 2019 mid-year population estimates were 5,463,300 and 3,152,900, respectively). We accessed complete coverage person-level datasets from Public Health Scotland (PHS) [27] and the Secure Anonymised Information Linkage (SAIL) Databank [28] in Wales. PHS receives individual-level data from all general or acute specialties in NHS hospitals in Scotland. The SAIL Databank receives linkable, routinely collected data from all NHS hospitals in Wales and 80% of general practices as well as other healthcare and administrative data.

We defined two primary outcome measures relating to severe COPD exacerbations: COPD-related emergency hospital admission, and death due to COPD. Emergency admissions for COPD were defined as those with a primary diagnosis of COPD recorded using the J43 and J44 codes of the 10th revision of the International Statistical Classification of Diseases (ICD-10). These data were extracted from the Scottish Morbidity Record 01 (SMR01) and the Patient Episodes Database for Wales (PEDW), both of which undergo regular data quality checks [29, 30]. In Wales, we also extracted the average length of stay (LOS) of COPD admissions.

Deaths due to COPD were defined as those with COPD (ICD-10 codes of J43 and J44) as the underlying cause of death in the National Records of Scotland (NRS) deaths database or the Annual District Death Extract (ADDE) in SAIL. Mortality data were regularly checked and validated by the UK Office for National Statistics (ONS) [31–33].

In Wales, we also had access to primary care and ED data. We defined COPD-related ED attendances as those with a COPD code (14B) as the primary diagnosis in the Emergency Department Dataset (EDDS) in SAIL. We defined a general practitioner-recorded AECOPD from the Welsh Longitudinal General Practice (WLGP) dataset in SAIL as an AECOPD code preceded by a COPD diagnosis code. The code sets are included in the supplementary information.

### Statistical analyses

We visualised the trends of the aforementioned outcomes for the first 30 calendar weeks in 2020 and corresponding national averages for the preceding 5 years. To investigate the impact of the UK lockdown on these outcomes, we undertook interrupted time series analyses with a single change point of the 23rd of March (week 13). We modelled the trends in the first 30 calendar weeks in 2020 and the corresponding 5-year averages (2015–2019) using Poisson generalised linear regression in R 4.0.3. The initial change point model in both the baseline period and 2020 had a pre-lockdown slope and intercept as well as an instantaneous change in intercept at the week of lockdown and a change in slope following lockdown. In the baseline period, we were anticipating no change in intercept and no change in slope at week 13. The final model was based upon the baseline and 2020 data and included a binary variable to differentiate the two periods, together with interaction terms for the slopes and instantaneous effects of lockdown. These interaction terms were used to compare the slopes prior to lockdown in the baseline period with 2020, to compare the instantaneous change in intercept at lockdown in baseline with 2020 and to compare the change in slope post-lockdown in baseline with 2020. Residual plots were used to check the linearity assumption, and the Breusch-Godfrey test [34] was used to assess autocorrelation. Separate models were used in Scotland and Wales, and *z* tests were used to compare the model coefficients between the two countries. We then used fixed-effect meta-analysis to derive pooled estimates from their weighted averages.

In pre-specified sensitivity analyses, we restricted the definition of the study outcomes in Wales to people aged at least 35 years with a smoking history (current or former smokers) documented in the Welsh Longitudinal General Practice (WLGP) dataset. Linkage to the WLGP dataset was available within the SAIL Databank through the Anonymised Linkage Field (ALF) [35]. We excluded records with missing ALF field or low-quality linkage.

For COPD admissions in Wales, we compared length of stay over the weeks 13 to 30 between 2020 and the 5-year average using a separate Poisson model controlled for week number. We used beta regression to model the relationship between the year of admission and the proportion of COPD admissions during which patients died due to COPD in every week, with week number as a covariate.

Data analysis was performed in R 4.0.2.

### Reporting guidelines

We followed the Framework for Enhanced Reporting of Interrupted Time Series (FERITS) [36] and the REporting of studies Conducted using Observational Routinely collected Data (RECORD) statement [37] in the reporting of this study (see the Supplementary Materials).

### Role of the funding source

The funders had no role in the study design, data collection and analysis, interpretation of findings, writing of the manuscript, or the decision to submit this manuscript for publication.

## Results

### Emergency admissions

**Fig. 1:**
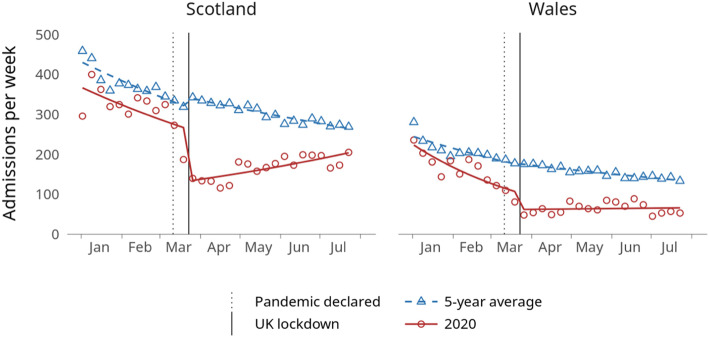
Weekly count of emergency COPD admissions in Scotland and Wales in 2020 and the corresponding 5-year averages (2015–2019, points) in addition to modelled trend lines

In the first 30 weeks of 2020, there were a total of 9847 emergency admissions for COPD in Scotland (6786) and Wales (3061). Those admissions were consistently lower than the averages of the corresponding periods in the preceding 5 years in both countries (Fig. 1).

In Scotland, the slope before lockdown in 2020 was decreasing (IRR 0.97, 95% CI 0.96 to 0.98), although there was no evidence that this slope differed from the 5-year average (IRR 1.00, 95% CI 0.98 to 1.01). Introducing lockdown in week 13 was associated with a reduction in admissions by 48% (IRR 0.52; 95% CI 0.47 to 0.58). This reduction was 52% greater than the average change at the same point in the preceding 5 years (IRR 0.48; 95% CI 0.42 to 0.55). The slope of admissions then gradually increased over the coming weeks (IRR 1.05, 95% CI 1.04 to 1.07), which was in contrast to the slope of the 5-year average which was decreasing (Fig. 1 and Table 1).

**Table 1:** Poisson models of emergency COPD admissions in 2020 and the corresponding 5-year averages (2015-2019).

	Scotland		Wales		Pooled estimates	
	IRR (95% CI)	*p* value	IRR (95% CI)	*p* value	IRR (95% CI)	*p* value
Pre-lockdown intercept in 2020 compared to 5-year average	0.83 (0.75, 0.91)	< 0.001	0.59 (0.51, 0.67)	< 0.001	0.74 (0.68, 0.80)	< 0.001
Slope in weeks 1–12						
5-year average	0.97 (0.97, 0.98)	< 0.001	0.97 (0.96, 0.98)	< 0.001	0.97 (0.97, 0.98)	< 0.001
2020	0.97 (0.96, 0.98)	< 0.001	0.94 (0.92, 0.95)	< 0.001	0.96 (0.95, 0.97)	< 0.001
2020 relative to 5-year average	1.00 (0.98, 1.01)	0.686	0.96 (0.95, 0.98)	< 0.001	0.99 (0.98, 1.00)	0.006
Change in level at week 13						
5-year average	1.08 (1.00, 1.18)	0.056	1.02 (0.92, 1.15)	0.666	1.06 (0.99, 1.13)	0.072
2020	0.52 (0.47, 0.58)	< 0.001	0.62 (0.53, 0.72)	< 0.001	0.55 (0.50, 0.60)	< 0.001
2020 relative to 5-year average	0.48 (0.42, 0.55)	< 0.001	0.60 (0.50, 0.73)	< 0.001	0.52 (0.46, 0.58)	< 0.001
Change in slope after week 13						
5-year average	1.01 (1.00, 1.02)	0.023	1.01 (1.00, 1.03)	0.034	1.01 (1.00, 1.02)	0.002
2020	1.05 (1.04, 1.07)	< 0.001	1.07 (1.05, 1.09)	< 0.001	1.06 (1.05, 1.07)	< 0.001
2020 relative to 5-year average	1.04 (1.03, 1.06)	< 0.001	1.06 (1.03, 1.08)	< 0.001	1.05 (1.03, 1.06)	< 0.001

In Wales, admissions in the first 12 weeks of 2020 were falling (IRR 0.94, 95% CI 0.92 to 0.95), slightly faster than the corresponding 5-year average (IRR 0.96, 95% CI 0.95 to 0.98). Introducing lockdown in week 13 was associated with a 38% reduction in admissions (IRR 0.62, 95% CI 0.53 to 0.72). This reduction was 40% greater than the average change at the same point in the preceding 5 years (IRR 0.60; 95% CI 0.50 to 0.73). Similar to the pattern seen in Scotland, there was a slight gradual increase in the slope of admissions over the ensuing weeks (IRR 1.07, 95% CI 1.05 to 1.09), which contrasted with the decreasing slope in the 5-year average (Fig. 1 and Table 1). Admissions for COPD were 22% shorter in 2020 during lockdown than in the corresponding periods in the preceding 5 years (IRR 0.78, 95% CI 0.78 to 0.79, *p* value < 0.001). However, there was no evidence that the proportion of COPD admissions during which patients died due to COPD in the weeks 13 to 30 was different in 2020 than the corresponding periods of the preceding 5 years (odds ratio 1.05, 95% CI 0.77 to 1.43, *p* value 0.756).

The estimated effects in Wales did not change significantly after restricting the analysis to those aged ≥ 35 years at admission with a smoking history (Table B, Additional file 1).

There was a 48% pooled reduction in admissions during lockdown across Scotland and Wales (IRR 0.52, 95% CI 0.46 to 0.58).

### Deaths

There were a total of 2554 deaths with COPD as the underlying cause in Scotland (1535) and Wales (1019) in the first 30 weeks of 2020.

**Fig. 2:**
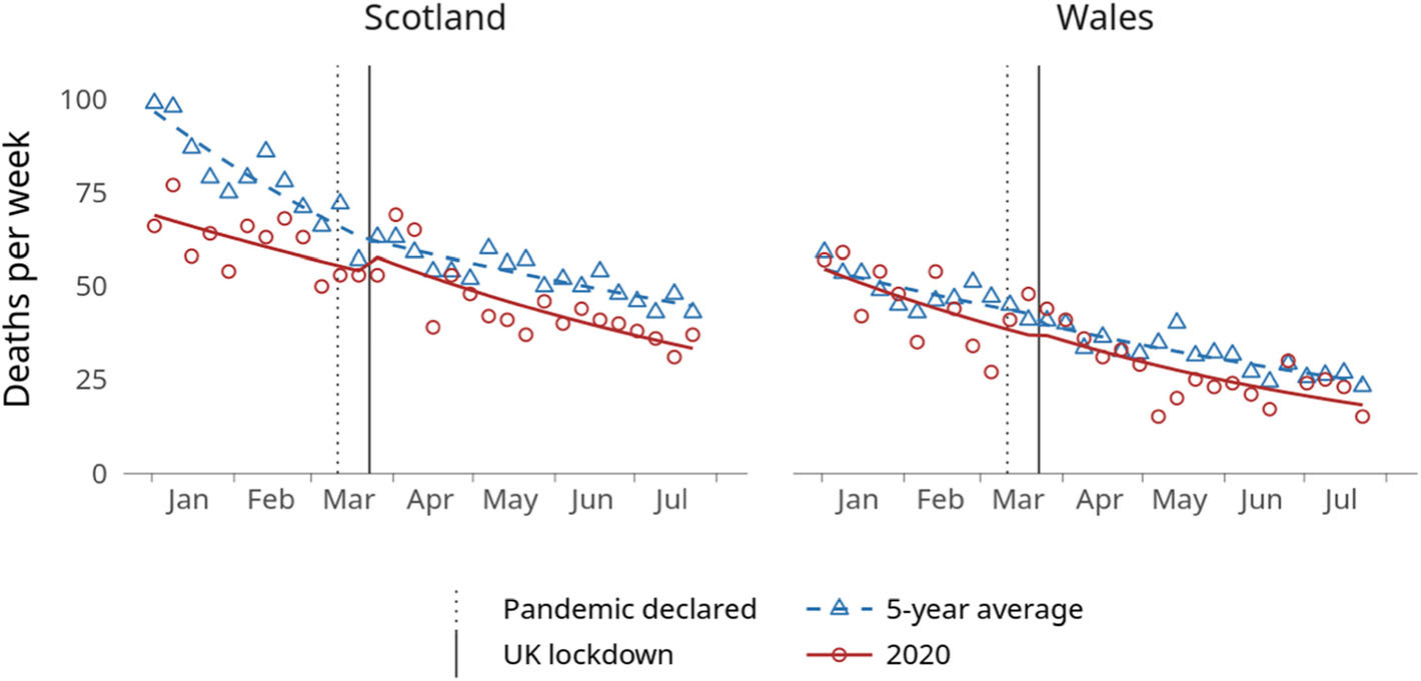
Weekly count of deaths with COPD as the underlying cause in Scotland and Wales in 2020 and the corresponding 5-year averages (2015–2019, points) in addition to modelled trend lines

In 2020 before lockdown, the trend of deaths with COPD as the underlying cause was not significantly different from the 5-year average (Fig. 2). The slope of deaths during this period was falling slightly in both nations (Scotland: IRR 0.98, 95 %CI 0.96 to 1.00; Wales: IRR 0.97, 95% CI 0.94 to 0.99, Table 2), as it did in the corresponding 5-year average.

**Table 2:** Poisson models of deaths with COPD as the underlying cause in 2020 and the corresponding 5-year averages (2015–2019).

	Scotland		Wales		Pooled estimates	
	IRR (95% CI)	*p* value	IRR (95% CI)	*p* value	IRR (95% CI)	*p* value
Pre-lockdown intercept in 2020 compared to 5-year average	0.87 (0.70, 1.08)	0.206	0.85 (0.66, 1.11)	0.234	0.86 (0.73, 1.02)	0.083
Slope in weeks 1–12						
5-year average	0.96 (0.94, 0.98)	< 0.001	0.98 (0.96, 1.00)	0.063	0.97 (0.95, 0.98)	< 0.001
2020	0.98 (0.96, 1.00)	0.042	0.97 (0.94, 0.99)	0.004	0.97 (0.96, 0.99)	0.001
2020 relative to 5-year average	1.02 (0.99, 1.05)	0.240	0.99 (0.95, 1.02)	0.446	1.00 (0.98, 1.03)	0.673
Change in level at week 13						
5-year average	1.02 (0.84, 1.22)	0.869	0.95 (0.75, 1.20)	0.657	0.99 (0.86, 1.14)	0.883
2020	1.09 (0.89, 1.33)	0.417	1.03 (0.81, 1.32)	0.803	1.06 (0.91, 1.24)	0.432
2020 relative to 5-year average	1.07 (0.81, 1.41)	0.627	1.09 (0.78, 1.53)	0.627	1.08 (0.87, 1.33)	0.494
Change in slope after week 13						
5-year average	1.02 (1.00, 1.04)	0.084	0.99 (0.97, 1.02)	0.687	1.01 (0.99, 1.03)	0.266
2020	0.99 (0.97, 1.01)	0.411	0.99 (0.96, 1.02)	0.699	0.99 (0.97, 1.01)	0.379
2020 relative to 5-year average	0.97 (0.94, 1.00)	0.078	1.00 (0.96, 1.04)	0.997	0.98 (0.96, 1.01)	0.169

There was no statistically significant change in deaths following the introduction of lockdown at week 13 of 2020 nor at the same point in the 5-year average in both nations (in Scotland, IRR in 2020 relative to 5-year average: 1.07, 95% CI 0.81 to 1.41; in Wales, IRR 1.09, 95% CI 0.78 to 1.53; pooled IRR: 1.08, 95% CI 0.87 to 1.33).

Deaths during the lockdown continued to fall, similar to the same period in the 5-year average (Fig. 2).

The estimated effects in Wales did not change after restricting the analysis to those with a smoking history who died at the age of ≥ 35 years (Table C, Additional file 1).

### Primary care consultations

**Fig. 3:**
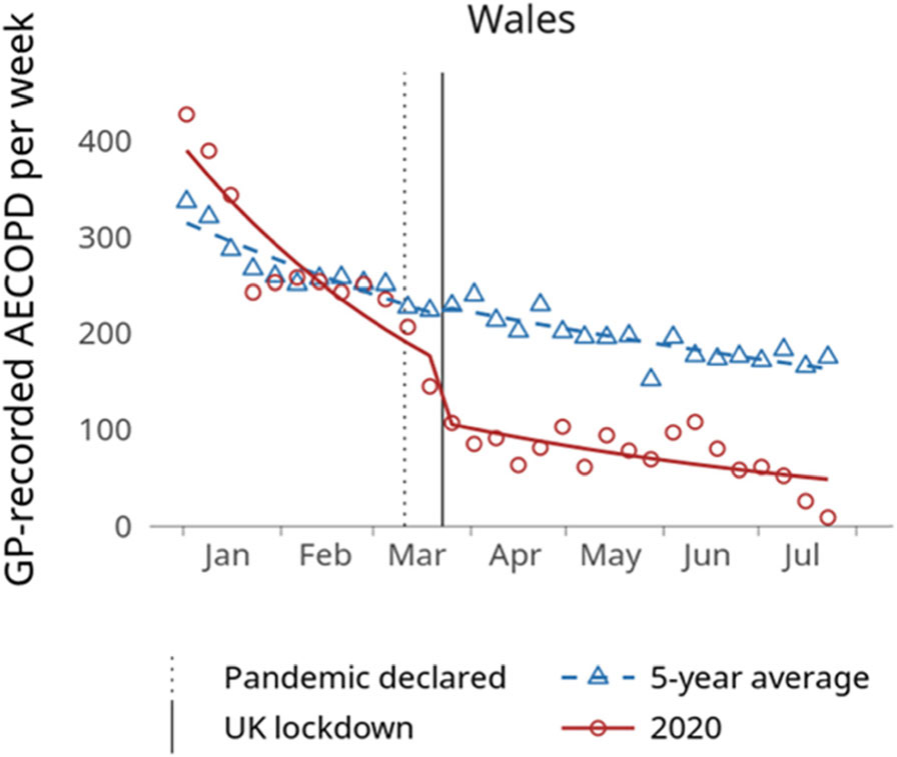
Weekly count of primary care consultations for acute exacerbation of COPD in Wales in 2020 and the corresponding 5-year averages (2015–2019, points) in addition to modelled trend lines

In Wales, there were 4575 primary care consultations for AECOPD in the first 30 weeks of 2020. Before lockdown, consultations were declining (IRR 0.93, 95% CI 0.92 to 0.94) in a steeper slope than the 5-year average (IRR 0.96, 95% CI 0.95 to 0.97, Table 3 and Fig. 3). Introducing lockdown was associated with a 39% instantaneous reduction in those consultations after adjusting for the 5-year averages (IRR 0.61, 95% CI 0.52 to 0.71). After week 13 of 2020, the slope continued to decline although it was less steep than the pre-lockdown slope (IRR for change in slope: 1.03, 95% CI 1.01 to 1.04). The estimated effects did not change after restricting the analysis to those with a smoking history who died at the age of ≥ 35 years (Table D, Additional file 1).

**Table 3:** Poisson models of primary care consultations for acute exacerbations of COPD in Wales in 2020 and the corresponding 5-year averages (2015–2019)

	IRR (95% CI)	*p* value
Pre-lockdown intercept in 2020 compared to 5-year average	0.76 (0.68, 0.86)	< 0.001
Slope in weeks 1–12		
5-year average	0.97 (0.96, 0.98)	< 0.001
2020	0.93 (0.92, 0.94)	< 0.001
2020 relative to 5-year average	0.96 (0.95, 0.97)	< 0.001
Change in level at week 13		
5-year average	1.05 (0.95, 1.16)	0.322
2020	0.64 (0.56, 0.72)	< 0.001
2020 relative to 5-year average	0.61 (0.52, 0.71)	< 0.001
Change in slope after week 13		
5-year average	1.01 (1.00, 1.02)	0.043
2020	1.03 (1.01, 1.04)	< 0.001
2020 relative to 5-year average	1.01 (1.00, 1.03)	0.147

### Emergency department attendances

**Fig. 4:**
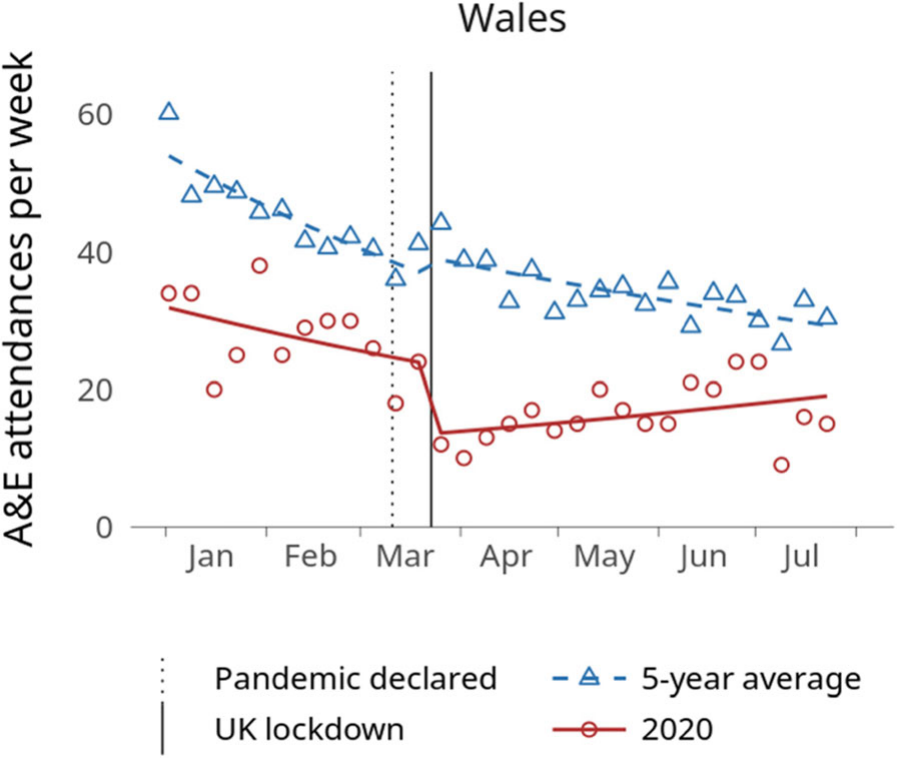
Weekly count of COPD-related emergency department attendances in Wales in 2020 and the corresponding 5-year averages (2015–2019, points) in addition to modelled trend lines

In Wales, there were 625 COPD-related ED attendances in the first 30 weeks of 2020. These events were consistently lower in the weeks 1 to 30 in 2020 than in the same period of the preceding 5 years (Fig. 4). There was no clear trend before lockdown (IRR 0.97, 95% CI 0.94 to 1.01, Table 4), unlike the corresponding slope in the baseline period which was decreasing. Introducing lockdown was associated with a 46% fall in attendances compared with the 5-year averages (IRR 0.54, 95% CI 0.36 to 0.81). After week 13 of 2020, there was an upward slope (IRR for change in slope: 1.05, 95% CI 1.01 to 1.09), unlike the corresponding slope in the baseline period which continued to decrease. The estimated effects did not change after restricting the analysis to those with a smoking history who died at the age of ≥ 35 years (Table E, Additional file 1).

**Table 4:** Poisson models of COPD-related emergency department attendances in Wales in 2020 and the corresponding 5-year averages (2015–2019)

	IRR (95% CI)	*p* value
Pre-lockdown intercept in 2020 compared to 5-year average	0.65 (0.48, 0.88)	0.006
Slope in weeks 1–12		
5-year average	0.97 (0.94, 0.99)	0.006
2020	0.97 (0.94, 1.01)	0.101
2020 relative to 5-year average	1.01 (0.97, 1.05)	0.686
Change in level at week 13		
5-year average	1.08 (0.85, 1.38)	0.508
2020	0.59 (0.42, 0.82)	0.002
2020 relative to 5-year average	0.54 (0.36, 0.81)	0.003
Change in slope after week 13		
5-year average	1.02 (0.99, 1.05)	0.227
2020	1.05 (1.01, 1.09)	0.019
2020 relative to 5-year average	1.03 (0.98, 1.08)	0.256

## Discussion

Our national level interrupted time-series analyses of the impact of lockdown across Scotland and Wales found substantial reductions in severe AECOPD leading to ED attendance and/or hospital admission as well as the less severe AECOPD that are recorded in primary care. The levels remained well below the corresponding 5-year averages throughout the study period. The easing of strict lockdown restrictions announced at the end of May 2020 [38, 39] did not lead to a substantial rebound in these events, but there was a gradual rise in ED attendances and admissions. There was no evidence of an increase in deaths due to COPD during the lockdown.

These findings are especially significant given that COPD exacerbations are one of the commonest reasons for emergency admission to hospital [40]. Reduced incidence of AECOPDs therefore increases healthcare capacity and resources available for those with COVID-19, as well as conferring obvious benefits to people with COPD. A reduction in AECOPD admissions is also particularly advantageous given the specific challenges in the hospital management of AECOPD during the pandemic—components of care, such as non-invasive ventilation (NIV), are associated with increased risk of viral transmission due to aerosolization [41] and can therefore only be delivered in specified clinical areas.

To the best of our knowledge, this is the first national level analysis of the impact of the COVID-19 lockdown on AECOPD incidence and mortality, using data across primary and secondary care as well as data on deaths. We used population-based data with high-to-complete geographical coverage across Scotland and Wales, which enabled comparison of findings between the two UK nations, which were broadly comparable.

Our study has some limitations. Firstly, there are no validated case definitions for COPD admissions or deaths in UK data, and so the case definitions used may have variable accuracy. We did not include deaths with COPD as a contributing cause because they would not have been specific enough for the purpose of this study. Data on AECOPD in primary care and ED data is under-recorded [42, 43]. However, our sensitivity analysis, limiting data to those aged ≥ 35 and ever-smokers, did not impact our overall results. Further, given that we were interested in trends over time rather than absolute numbers and that coding practices of these events are unlikely to have changed, this is unlikely to have significantly affected our findings.

The observed reduction in AECOPDs during lockdown does not necessarily imply direct causal effects of lockdown. Nonetheless, they are likely to have been mediated by reductions in the transmission of other respiratory pathogens and outdoor air pollution during lockdown [7, 8, 14], both of which have a major role in triggering AECOPD [44]. These factors could also have mediated the reductions in emergency admissions for asthma that have been reported following lockdowns [45–48].

A number of potential confounding factors such as changes in prescribing for COPD [49], behavioural changes related to improved self-management and smoking reduction/cessation [50], and possibly improved air quality [7] during the first wave of pandemic might have contributed to the observed reduction in AECOPDs. However, we were not in a position to adjust for these potential sources of bias. Care therefore needs to be taken when interpreting our findings. Furthermore, national messaging on the need to avoid overwhelming the NHS and the fear about the spread of SARS-CoV-2 might have contributed to the fall in AECOPD-related ED attendances and admissions during the first wave of the pandemic. However, this effect on healthcare-seeking behaviour is likely limited in those experiencing a severe AECOPD, which usually requires hospital assessment and treatment. In addition, the corresponding decline in AECOPD in primary care records suggests a true decline in incidence during lockdown. However, it is possible that the milder forms of AECOPDs have been self-managed by patients and were not presented to the health care system during lockdown.

The lifting of restrictions on travel and social contact was associated with a gradual rise in emergency admissions for AECOPD seen in our data for Scotland and Wales. This could be partly due to an increase in the circulation of respiratory viruses (mainly rhinoviruses initially) [8] and levels of outdoor air pollutants [51]. However, this could also be explained by an increasing threshold of hospital admission, since primary care consultations for AECOPD continued to fall towards summer in accordance with the typical seasonal trend of AECOPD in the UK [52].

Although there is extensive literature on COPD as a risk factor for COVID-19 severe outcomes and deaths [53–55], there is currently limited data examining how the pandemic, and specifically lockdowns, has affected COPD deaths more widely. Two studies from Hong Kong and England reported no difference in inpatient mortality during admissions for COPD exacerbation [13, 15]. Analysis of excess mortality from specific conditions is important in understanding whether reductions in emergency healthcare utilisation represent a true reduction in incidence or avoidance of healthcare settings, the latter of which could lead to increased mortality. Our study has shown no significant increase in non-COVID COPD deaths over the first 30 weeks of 2020. This is in contrast to a recent analysis of cardiovascular mortality in England and Wales [3], which demonstrated excess non-COVID acute cardiovascular deaths. The authors suggested that people with these diagnoses either did not seek help for their illness or were not referred to hospital, consistent with the greatest proportional increase in cardiovascular deaths occurring in community settings. Data from England from March to September 2020 from the Office for National Statistics shows that COVID-19 accounted for over 90% of excess deaths among those aged over 75 from both sexes, but the proportion of non-COVID excess deaths was higher across younger people [56]. The leading causes of these non-COVID excess deaths were dementia, ischaemic heart disease, cerebrovascular disease and other circulatory diseases. Deaths in England due to “chronic lower respiratory diseases” (ICD-10 codes J40-47) including COPD actually fell compared to expected levels when assessed cumulatively from March to September 2020 [56], consistent with our findings in Scotland and Wales.

There are several important areas for future investigation to understand underlying reasons for our findings. These include person-level analyses of how factors related to COPD, such as disease severity, control and health service utilisation and positive drivers such as reduced exposure to respiratory pathogens and pollutants, improved self-management, smoking cessation and other behavioural changes, might have affected the risk of AECOPD and related death during lockdown. If further work suggests that altered outdoor air pollution levels have played a significant role, findings should spur increased drive to improve air quality longer term [57]. Other interventions with the potential to produce lasting reductions in the rate of severe AECOPD include the facilitation of self-management of chronic conditions and the consolidation of public health messages to reduce the transmission of respiratory infections, including hand hygiene, use of facemasks and wider deployment of testing and isolation when viruses are most likely to be circulating.

## Conclusions

We found significant declines in AECOPDs across primary and secondary care in Scotland and Wales during the initial UK-wide lockdown in 2020, with no associated increase in non-COVID deaths due to COPD. Our study strengthens the notion that outcomes relating to chronic respiratory disease improved during the lockdown period. It is crucial to assess the fuller impact of the pandemic on care and outcomes in chronic health conditions such as COPD, including non-COVID related morbidity and mortality. This will inform the targeting of public health strategy to minimise any adverse effects as well as capture any positive elements that could be harnessed to reduce hospital admissions in vulnerable groups over the longer term.

## Supplementary Information

**Additional file 1.** Includes Read codes for COPD diagnosis, acute exacerbation of COPD, and smoking status used for sensitivity analysis, model diagnostics (Table A), the results of the sensitivity analysis (Tables B, C, D, and E), and the reporting checklists (FERITS, STROBE, RECORD).

## Data Availability

The anonymised person-level data supporting the conclusions of this article are held by Public Health Scotland (https://publichealthscotland.scot/) and the SAIL Databank (https://saildatabank.com/) and are restricted and not publicly available but can be accessed upon reasonable requests and with permission from PHS and SAIL. All proposals to use SAIL are carefully reviewed by an independent Information Governance Review Panel (IGRP) to ensure proper and appropriate use of data (https://www.saildatabank.com/application-process). When approved, access is then provided through the SAIL Gateway, a privacy-protecting safe haven and a secure remote access system.

## Abbreviations

ADDE: Annual District Death Extract
AECOPD: Acute exacerbation of chronic obstructive pulmonary disease
ALF: Anonymous Linking Field
CI: Confidence interval
COPD: Chronic obstructive pulmonary disease
COVID-19: Coronavirus disease 2019
ED: Emergency department
EDDS: Emergency Department Dataset
FERITS: Framework for Enhanced Reporting of Interrupted Time Series
ICD: International Statistical Classification of Diseases
IRR: Incidence rate ratio
LOS: Length of stay
NHS: National Health Service
NIV: Non-invasive ventilation
NRS: National Records of Scotland
ONS: Office for National Statistics
PEDW: Patient Episode Database for Wales
PHS: Public Health Scotland
RECORD: REporting of studies Conducted using Observational Routinely-collected Data
SAIL: Secure Anonymised Information Linkage
SARS-CoV-2: Severe acute respiratory syndrome coronavirus 2
SMR: Scottish Morbidity Records
UK: United Kingdom
WLGP: Welsh Longitudinal General Practice
