# Supplementary material for: Impact of COVID-19 lockdown on the incidence and mortality of acute exacerbations of chronic obstructive pulmonary disease: national interrupted time series analyses for Scotland and Wales
Source: BMC Med. 2021 May 17;19:124. doi: 10.1186/s12916-021-02000-w (PMC8126470; doi:10.1186/s12916-021-02000-w)
Supplement: Supplementary file 1 — Additional file 1. Includes Read codes for COPD diagnosis, acute exacerbation of COPD, and smoking status used for sensitivity analysis, model diagnostics (Table A), the results of the sensitivity analysis (Tables B, C, D, and E), and the reporting checklists (FERITS, STROBE, RECORD). [file 12916_2021_2000_MOESM1_ESM.docx]

# Additional file 1

### Code sets

#### COPD diagnosis

| H3... | Chronic obstructive pulmonary disease |
| --- | --- |
| H31.. | Chronic bronchitis |
| H310. | Simple chronic bronchitis |
| H3100 | Chronic catarrhal bronchitis |
| H310z | Simple chronic bronchitis NOS |
| H311. | Mucopurulent chronic bronchitis |
| H3110 | Purulent chronic bronchitis |
| H3111 | Fetid chronic bronchitis |
| H311z | Mucopurulent chronic bronchitis NOS |
| H312. | Obstructive chronic bronchitis |
| H3120 | Chronic asthmatic bronchitis |
| H3121 | Emphysematous bronchitis |
| H3123 | Bronchiolitis obliterans |
| H312z | Obstructive chronic bronchitis NOS |
| H313. | Mixed simple and mucopurulent chronic bronchitis |
| H31y. | Other chronic bronchitis |
| H31y1 | Chronic tracheobronchitis |
| H31yz | Other chronic bronchitis NOS |
| H31z. | Chronic bronchitis NOS |
| H32.. | Emphysema |
| H320. | Chronic bullous emphysema |
| H3200 | Segmental bullous emphysema |
| H3201 | Zonal bullous emphysema |
| H3202 | Giant bullous emphysema |
| H3203 | Bullous emphysema with collapse |
| H320z | Chronic bullous emphysema NOS |
| H321. | Panlobular emphysema |
| H322. | Centrilobular emphysema |
| H32y. | Other emphysema |
| H32y0 | Acute vesicular emphysema |
| H32y1 | Atrophic (senile) emphysema |
| H32y2 | MacLeod's unilateral emphysema |
| H32yz | Other emphysema NOS |
| H32z. | Emphysema NOS |
| H36.. | Mild chronic obstructive pulmonary disease |
| H37.. | Moderate chronic obstructive pulmonary disease |
| H38.. | Severe chronic obstructive pulmonary disease |
| H39.. | Very severe chronic obstructive pulmonary disease |
| H3A.. | End stage chronic obstructive airways disease |
| H3B.. | Asthma-chronic obstructive pulmonary disease overlap syndrome |
| H3y.. | Other specified chronic obstructive airways disease |
| H3z.. | Chronic obstructive airways disease NOS |
| H4640 | Chronic emphysema due to chemical fumes |
| Hyu30 | [X]Other emphysema |
| Hyu31 | [X]Other specified chronic obstructive pulmonary disease |

#### Acute exacerbation of COPD

| H3122 | Acute exacerbation of chronic obstructive airways disease |
| --- | --- |
| H3Y1. | Chronic obstructive pulmonary disease with acute exacerbation; unspecified |

#### Smoking history

| 137.. | Tobacco consumption |
| --- | --- |
| 1372. | Trivial smoker - < 1 cig/day |
| 1373. | Light smoker - 1-9 cigs/day |
| 1374. | Moderate smoker - 10-19 cigs/d |
| 1375. | Heavy smoker - 20-39 cigs/day |
| 1376. | Very heavy smoker - 40+cigs/d |
| 1377. | Ex-trivial smoker (<1/day) |
| 1378. | Ex-light smoker (1-9/day) |
| 1379. | Ex-moderate smoker (10-19/day) |
| 137a. | Pipe tobacco consumption |
| 137A. | Ex-heavy smoker (20-39/day) |
| 137b. | Ready to stop smoking |
| 137B. | Ex-very heavy smoker (40+/day) |
| 137c. | Thinking about stop smoking |
| 137C. | Keeps trying to stop smoking |
| 137d. | Not interested in stop smoking |
| 137D. | Admitted tobacco cons untrue ? |
| 137e. | Smoking restarted |
| 137f. | Reason for restarting smoking |
| 137F. | Ex-smoker - amount unknown |
| 137g. | Cigarette pack-years |
| 137G. | Trying to give up smoking |
| 137h. | Min from wake to 1st tobac con |
| 137H. | Pipe smoker |
| 137j. | Ex-cigarette smoker |
| 137J. | Cigar smoker |
| 137k. | Refusal to give smoking status |
| 137K. | Stopped smoking |
| 137K0 | Recently stopped smoking |
| 137l. | Ex roll-up cigarette smoker |
| 137m. | Failed attempt to stop smoking |
| 137M. | Rolls own cigarettes |
| 137n. | Total time smoked |
| 137N. | Ex pipe smoker |
| 137o. | Waterpipe tobacco consumption |
| 137O. | Ex cigar smoker |
| 137P. | Cigarette smoker |
| 137Q. | Smoking started |
| 137R. | Current smoker |
| 137S. | Ex smoker |
| 137T. | Date ceased smoking |
| 137V. | Smoking reduced |
| 137X. | Cigarette consumption |
| 137Y. | Cigar consumption |
| 137Z. | Tobacco consumption NOS |
| 13p.. | Smoking cessation milestones |
| 13p0. | Negotiatd date cessatn smoking |
| 13p5. | Smoking cessn progm start date |
| 13p50 | Practice smok cess pr strt dat |
| 13p8. | Lost to smok cessation fllw-up |
| 6791. | Health ed. - smoking |
| 67910 | Health ed. - parental smoking |
| 67H1. | Lifestyle adv re smoking |
| 67H6. | Brf intervention smoking cessn |
| 745H. | Smoking cessation therapy |
| 745H0 | Nic rep thera us nicot ptches |
| 745H1 | Nicot repl thera us nicot gum |
| 745H2 | Nic repl thera us nic inhalatr |
| 745H3 | Nic repl thera us nic lozenges |
| 745H4 | Smoking cessation drug therapy |
| 745H5 | Varenicline therapy |
| 745Hy | OS smoking cessation therapy |
| 745Hz | Smoking cessation therapy NOS |
| 8B2B. | Nicotine replacement therapy |
| 8B2B0 | Issue nictn replce ther vouch |
| 8B31G | Varenicline smok cess th offer |
| 8B3f. | Nicotine replacemt therap free |
| 8B3Y. | Over countr nicotine repl ther |
| 8BP3. | Nic rep thera prov by com phar |
| 8CAg. | Smoke cess advi prov com pharm |
| 8CAL. | Smoking cessation advice |
| 8CdB. | Stop smok serv opport signpost |
| 8H7i. | Referral: smok cessatn advisor |
| 8HBM. | Stop smoking fce to fce flw-up |
| 8HBP. | Smoking cesn 12 week follow-up |
| 8HkQ. | Refer to NHS stop smoking srvc |
| 8HTK. | Referl to stop-smoking clinic |
| 8I2I. | Nicotin replace ther contraind |
| 8I39. | Nicotine repl therapy refused |
| 8IAj. | Smok cessation advice declined |
| 8IEK. | Smok cessation program declned |
| 8IEM. | Smoking cess drug therapy decl |
| 8IEM0 | Varenicline smok cess th decln |
| 8IEo. | Ref smoking cessn serv declnd |
| 8T08. | Ref to smoking cessn service |
| 9kc.. | Smoking cessation - enh se adm |
| 9kc0. | Smok ces templt completd - ESA |
| 9kf1. | Ref COPD stru smok asses - ESA |
| 9kf2. | COPD stru smok ass decld - ESA |
| 9km.. | Ex-smoker annual review - ESA |
| 9ko.. | Currnt smokr annua reviw - ESA |
| 9N2k. | Seen by smoking cesstn advisor |
| 9N4M. | DNA - Smoking cessation clinic |
| 9Ndf. | Con giv fr flw-up by smk ces t |
| 9Ndg. | Dec con f flw up by smk cess t |
| 9NdV. | Con giv fw-up aft smo ces inte |
| 9NdW. | Con giv for smkg cess data shr |
| 9NdY. | Dec con fol eval smo ces inter |
| 9NdZ. | Dec con for smok cess data sha |
| 9NS02 | Ref smoking cessat serv offer |
| 9OO.. | Anti-smoking monitoring admin. |
| 9OO1. | Attends stop smoking monitor. |
| 9OO2. | Refuses stop smoking monitor |
| 9OO3. | Stop smoking monitor default |
| 9OO4. | Stop smoking monitor 1st lettr |
| 9OO5. | Stop smoking monitor 2nd lettr |
| 9OO6. | Stop smoking monitor 3rd lettr |
| 9OO7. | Stop smoking monitor verb.inv. |
| 9OO8. | Stop smoking monitor phone inv |
| 9OO9. | Stop smoking monitoring delete |
| 9OOA. | Stop smoking monitor.chck done |
| 9OOB. | Stop smokg invtn SMS txt mssge |
| 9OOB0 | Stop smoking ivtn 1st txt msge |
| 9OOB1 | Stop smoking ivtn 2nd txt msge |
| 9OOB2 | Stop smoking ivtn 3rd txt msge |
| 9OOZ. | Stop smoking monitor admin.NOS |
| du3.. | NICOTINE |
| du31. | NICOTINE 2mg chewing gum |
| du32. | NICOTINE 4mg chewing gum |
| du33. | NICORETTE 2mg chewing gum |
| du34. | NICORETTE 4mg chewing gum |
| du35. | NICOTINELL TTS 10 patches |
| du36. | NICOTINELL TTS 20 patches |
| du37. | NICOTINELL TTS 30 patches |
| du38. | NICOTINE 7mg/24hours patches |
| du39. | NICOTINE 14mg/24hours patches |
| du3a. | NICORETTE nasal spray |
| du3A. | NICOTINE 21mg/24hours patches |
| du3b. | NICOTINE 10mg/mL nasal spray |
| du3B. | *NICORETTE 5mg patches x7 |
| du3c. | NICOTINELL ORIGINAL 2mg gum |
| du3C. | *NICORETTE 10mg patches x7 |
| du3d. | NICOTINELL MINT 2mg gum |
| du3D. | *NICORETTE 15mg patches x7 |
| du3e. | *NICOTINE 10mg inhal start pck |
| du3E. | *NICORETTE 15mg patches x28 |
| du3f. | *NICOTINE 10mg inhal refill pk |
| du3F. | NICOTINE 5mg/16hours patches |
| du3g. | *NICORETT 10mg inhal start pck |
| du3G. | NICOTINE 10mg/16hours patches |
| du3h. | *NICORETT 10mg inhal refill pk |
| du3H. | NICOTINE 15mg/16hours patches |
| du3i. | NICOTINELL ORIGINAL 4mg gum |
| du3I. | NIQUITIN CQ 2mg original loz |
| du3j. | NICOTINELL MINT 4mg chew gum |
| du3J. | *NICABATE 7mg patches x14 |
| du3k. | NIQUITIN CQ 7mg/24hrs patches |
| du3K. | *NICABATE 14mg patches x14 |
| du3l. | NIQUITIN CQ 14mg/24hrs patches |
| du3L. | *NICABATE 21mg patches x14 |
| du3m. | NIQUITIN CQ 21mg/24hrs patches |
| du3M. | *NICABATE 7mg patches x7 |
| du3n. | NICOTINE 2mg s/l tabs |
| du3N. | *NICABATE 14mg patches x7 |
| du3o. | NICORETTE MICROTAB 2mg s/l tab |
| du3O. | NIQUITIN CQ 7mg/24hrs clr ptch |
| du3p. | *NICOTINE 1mg mint lozenges |
| du3P. | *NICABATE 21mg patches x7 |
| du3q. | NICOTINELL MINT 1mg lozenges |
| du3Q. | *NICORETTE 15mg patches x3 |
| du3r. | NIQUITIN CQ 14mg/24hr clr ptch |
| du3R. | *NICONIL-11 patches |
| du3s. | NIQUITIN CQ 21mg/24hr clr ptch |
| du3S. | *NICONIL-22 patches |
| du3t. | NICOTINE 2mg fruit chewing gum |
| du3T. | *NICOTINE 11mg/24hours patches |
| du3u. | NICOTINE 4mg fruit chewing gum |
| du3U. | *NICOTINE 22mg/24hours patches |
| du3v. | *NICOTINE 2mg citrus chew gum |
| du3V. | NICORETTE 2mg mint chewing gum |
| du3w. | *NICORETTE CITRUS 2mg chew gum |
| du3W. | NICORETTE MINT PLUS 4mg gum |
| du3x. | NICOTINE 1mg lozenges |
| du3X. | NICOTINE 2mg mint chewing gum |
| du3y. | NICOTINE 2mg lozenges |
| du3Y. | NICOTINE 4mg mint chewing gum |
| du3z. | NICOTINE 4mg lozenges |
| du3Z. | *NICONIL 22 starter pack |
| du6.. | BUPROPION |
| du61. | ZYBAN 150mg m/r tablets |
| du6z. | BUPROPION HCL 150mg m/r tabs |
| du7.. | NICOTINE 2 |
| du71. | NIQUITIN CQ 4mg original loz |
| du72. | NIQUITIN CQ 2mg mint chew gum |
| du73. | NIQUITIN CQ 4mg mint chew gum |
| du74. | *NICORETTE 15mg patches x2 |
| du75. | NICOTINELL 2mg liquorice gum |
| du76. | NICOTINELL 4mg liquorice gum |
| du77. | NICOTINELL 2mg mint lozenges |
| du78. | NIQUITIN CQ 2mg mint lozenges |
| du79. | NIQUITIN CQ 4mg mint lozenges |
| du7a. | *NICOTINELL ICEMINT 4mg gum |
| du7A. | NICORETTE FRESHMT 2mg chew gum |
| du7b. | NICORETTE 15mg inhalator |
| du7B. | NICORETTE FRESHMT 4mg chew gum |
| du7c. | NICOTINE 15mg inhalator |
| du7C. | *NICOTINELL CLASSIC 2mg gum |
| du7d. | NICASSIST 7mg/24hours patches |
| du7D. | *NICOTINELL CLASSIC 4mg gum |
| du7e. | NICASSIST 14mg/24hours patches |
| du7E. | NICORETTE FRESHFRUIT 2mg gum |
| du7f. | NICASSIST 21mg/24hours patches |
| du7F. | NICORETTE FRESHFRUIT 4mg gum |
| du7g. | NICORETTE COOLS 2mg lozenges |
| du7G. | *NICOPATCH 7mg/24hours patches |
| du7h. | NICORETTE COOLS 4mg lozenges |
| du7H. | *NICOPATCH 14mg/24hrs patches |
| du7i. | NIQUTN PREQUIT 21mg/24hrs ptch |
| du7I. | *NICOPATCH 21mg/24hrs patches |
| du7j. | NICORETTE FRUITFUSION 2mg gum |
| du7J. | *NICOPASS 1.5mg fresh mint loz |
| du7k. | NICORETTE FRUITFUSION 4mg gum |
| du7K. | *NICOPASS 1.5mg liquor mnt loz |
| du7l. | NICORETTE FRUITFUSION 6mg gum |
| du7L. | NIQUITIN PRE-QUIT 4mg mint loz |
| du7M. | NICORETTE INVISI 10mg patches |
| du7n. | NICOTINE 6mg fruit chewing gum |
| du7N. | NICORETTE INVISI 15mg patches |
| du7o. | NICOTINE 4mg icemint chew gum |
| du7O. | NICORETTE INVISI 25mg patches |
| du7p. | NICOTINE 2mg icemint chew gum |
| du7P. | NICORETTE ICY WHITE 2mg gum |
| du7q. | NICOTINE 1mg oromucosal spray |
| du7Q. | NICORETTE ICY WHITE 4mg gum |
| du7r. | NICOTINE 1.5mg cherry lozenges |
| du7R. | NIQUITIN MINIS MINT 1.5mg loz |
| du7s. | NICOTINE 4mg cherry lozenges |
| du7S. | NIQUITIN MINIS MINT 4mg loz |
| du7t. | *NICOTINE 15mg patch+2mg gum |
| du7T. | NICORETTE MIC LEM 2mg s/l tabs |
| du7u. | NICOTINE 1.5mg lozenges |
| du7U. | *NICORETTE COMBI patch+gum |
| du7v. | NICOTINE 25mg/16hours patches |
| du7V. | NIQUITIN MINIS 1.5mg chrry loz |
| du7w. | *NICOTINE 1.5mg fresh mint loz |
| du7W. | NIQUITIN MINIS 4mg cherry loz |
| du7x. | *NICOTINE 1.5mg liquor mnt loz |
| du7X. | *NICORETTE FRESHMINT 2mg loz |
| du7y. | NICOTINE 4mg liquorice gum |
| du7Y. | NICORETTE QUICKMIST 1mg spray |
| du7z. | NICOTINE 2mg liquorice gum |
| du7Z. | *NICOTINELL ICEMINT 2mg gum |
| du8.. | VARENICLINE |
| du81. | CHAMPIX 1mg tablets |
| du82. | CHAMPIX 500microgram tablets |
| du83. | CHAMPIX INITIATION pack |
| du8x. | VARENICLINE 500mcg+1mg tablets |
| du8y. | VARENICLINE 500microgram tabs |
| du8z. | VARENICLINE 1mg tablets |
| duB.. | NICOTINE 3 |
| duB1. | NIQUITIN STRIPS 2.5mg mint flm |
| duB2. | NIQUITIN MINIS 1.5mg ornge loz |
| duB3. | NICOTINELL SUPPORT ICE 2mg gum |
| duB4. | NICOTINELL SUPPORT ICE 4mg gum |
| duBz. | NICOTINE 2.5mg oral film |
| H3101 | Smokers' cough |
| H310z | Simple chronic bronchitis NOS |
| J0364 | Tobacco deposit on teeth |
| SMC.. | Toxic effect/tobacco+ nicotine |
| ZV116 | [V]PH of tobacco abuse |
| ZV4K0 | [V]Tobacco use |
| ZV6D8 | [V]Tobacco abuse counselling |

## Model diagnostics


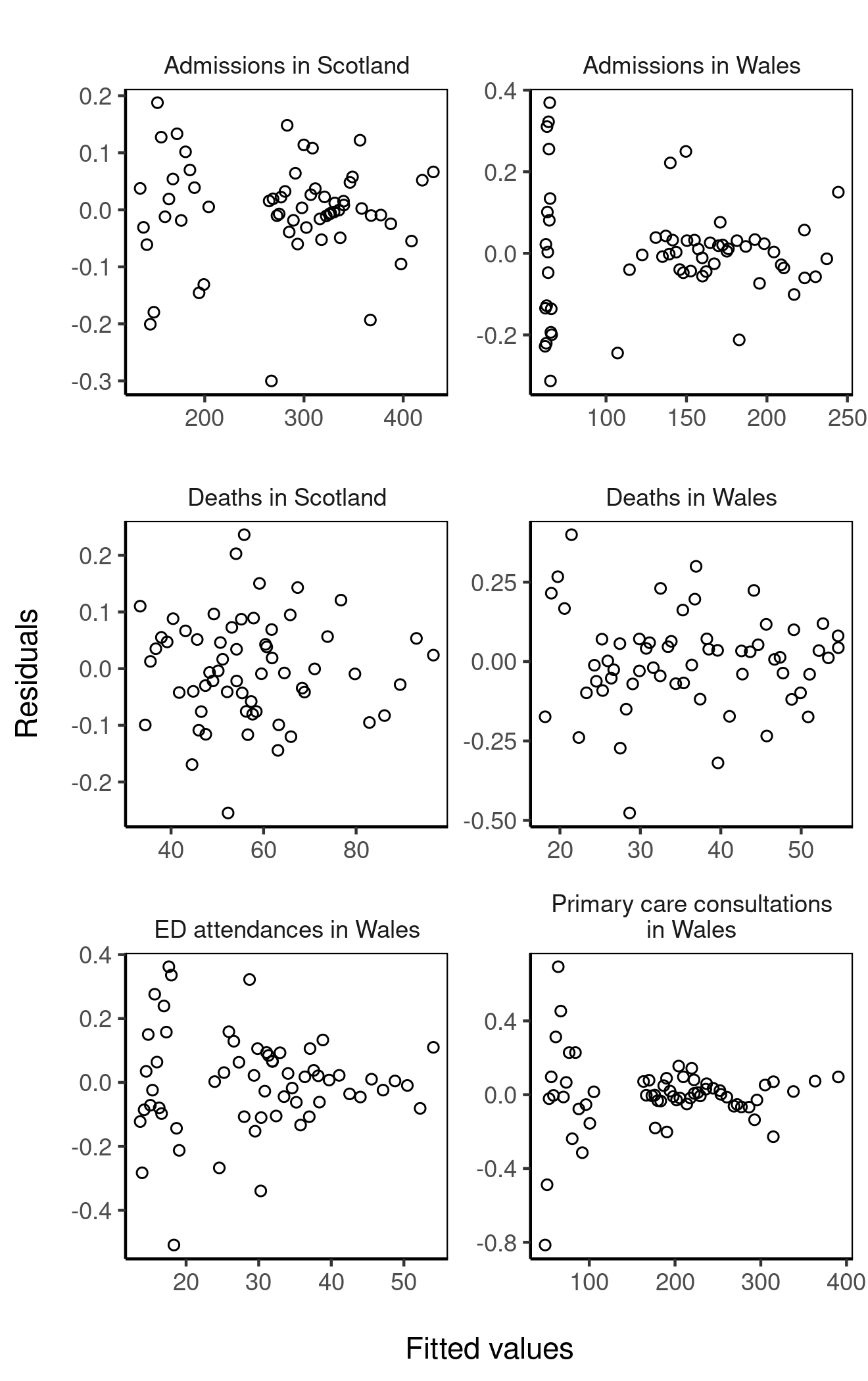


**Figure A**: Fitted values vs residuals.

**Table A:** Model diagnostics.

| **Country** | **Model** | **null.deviance** | **deviance** | **deviance.change** | **nobs** | **df.null** | **df.residual** | **logLik** | **AIC** | **BIC** |
| --- | --- | --- | --- | --- | --- | --- | --- | --- | --- | --- |
| Scotland | Admissions | 1640.4 | 110.8 | 93% | 60 | 59 | 52 | -277.8 | 571.5 | 588.3 |
| Wales | Admissions | 1570.6 | 95.9 | 94% | 60 | 59 | 52 | -247.9 | 511.8 | 528.5 |
| Scotland | Deaths | 232.8 | 27.4 | 88% | 60 | 59 | 52 | -189.3 | 394.7 | 411.4 |
| Wales | Deaths | 223.4 | 44.4 | 80% | 60 | 59 | 52 | -183.5 | 383.0 | 399.7 |
| Wales | ED attendances | 276.4 | 32.6 | 88% | 60 | 59 | 52 | -170.7 | 357.4 | 374.1 |
| Wales | Primary care consultations | 2916.0 | 202.8 | 93% | 60 | 59 | 52 | -308.4 | 632.7 | 649.5 |

## Sensitivity analyses

**Table B:** Poisson models of emergency COPD admissions at the age ≥ 35 years with a smoking history, in Wales in 2020 and the five-year average (2015-2019).

|  | IRR (95% CI) | p-value |
| --- | --- | --- |
| Pre-lockdown intercept in 2020 compared to 5-year average | 0.60 (0.52, 0.70) | <0.001 |
| Slope in weeks 1-12 |  |  |
| 5-year average | 0.97 (0.96, 0.98) | <0.001 |
| 2020 | 0.94 (0.93, 0.95) | <0.001 |
| 2020 relative to 5-year average | 0.97 (0.95, 0.98) | <0.001 |
| Change in level at week 13 |  |  |
| 5-year average | 1.02 (0.90, 1.15) | 0.742 |
| 2020 | 0.63 (0.53, 0.74) | <0.001 |
| 2020 relative to 5-year average | 0.62 (0.50, 0.76) | <0.001 |
| Change in slope after week 13 |  |  |
| 5-year average | 1.01 (1.00, 1.03) | 0.073 |
| 2020 | 1.07 (1.05, 1.09) | <0.001 |
| 2020 relative to 5-year average | 1.06 (1.03, 1.08) | <0.001 |

IRR: incidence rate ratio; CI: confidence interval.

**Table C:** Poisson models of deaths with COPD as the underlying cause at the age ≥ 35 years with smoking history in Wales in 2020 and the five-year average (2015-2019).

|  | IRR (95% CI) | p-value |
| --- | --- | --- |
| Pre-lockdown intercept in 2020 compared to 5-year average | 0.86 (0.65, 1.14) | 0.297 |
| Slope in weeks 1-12 |  |  |
| 5-year average | 0.98 (0.96, 1.01) | 0.213 |
| 2020 | 0.97 (0.94, 0.99) | 0.018 |
| 2020 relative to 5-year average | 0.98 (0.95, 1.02) | 0.411 |
| Change in level at week 13 |  |  |
| 5-year average | 0.90 (0.69, 1.16) | 0.400 |
| 2020 | 1.05 (0.80, 1.37) | 0.743 |
| 2020 relative to 5-year average | 1.17 (0.81, 1.69) | 0.412 |
| Change in slope after week 13 |  |  |
| 5-year average | 0.99 (0.96, 1.02) | 0.508 |
| 2020 | 0.99 (0.96, 1.02) | 0.500 |
| 2020 relative to 5-year average | 1.00 (0.96, 1.05) | 0.978 |

IRR: incidence rate ratio; CI: confidence interval.

**Table D:** Poisson models of primary care consultations for acute exacerbation of COPD among people aged ≥ 35 years with smoking history in Wales in 2020 and the five-year average (2015-2019).

|  | IRR (95% CI) | p-value |
| --- | --- | --- |
| Pre-lockdown intercept in 2020 compared to 5-year average | 0.77 (0.69, 0.87) | <0.001 |
| Slope in weeks 1-12 |  |  |
| 5-year average | 0.97 (0.96, 0.98) | <0.001 |
| 2020 | 0.93 (0.92, 0.94) | <0.001 |
| 2020 relative to 5-year average | 0.96 (0.95, 0.98) | <0.001 |
| Change in level at week 13 |  |  |
| 5-year average | 1.05 (0.95, 1.16) | 0.320 |
| 2020 | 0.64 (0.56, 0.73) | <0.001 |
| 2020 relative to 5-year average | 0.61 (0.52, 0.71) | <0.001 |
| Change in slope after week 13 |  |  |
| 5-year average | 1.01 (1.00, 1.03) | 0.037 |
| 2020 | 1.03 (1.01, 1.04) | 0.001 |
| 2020 relative to 5-year average | 1.01 (0.99, 1.03) | 0.202 |

IRR: incidence rate ratio; CI: confidence interval.

**Table E:** Poisson models of COPD-related emergency department attendances among people aged ≥ 35 years with smoking history in Wales in 2020 and the five-year average (2015-2019).

|  | IRR (95% CI) | p-value |
| --- | --- | --- |
| Pre-lockdown intercept in 2020 compared to 5-year average | 0.67 (0.48, 0.93) | 0.017 |
| Slope in weeks 1-12 |  |  |
| 5-year average | 0.97 (0.94, 0.99) | 0.008 |
| 2020 | 0.98 (0.94, 1.01) | 0.148 |
| 2020 relative to 5-year average | 1.01 (0.97, 1.06) | 0.615 |
| Change in level at week 13 |  |  |
| 5-year average | 1.07 (0.83, 1.39) | 0.605 |
| 2020 | 0.60 (0.42, 0.85) | 0.005 |
| 2020 relative to 5-year average | 0.56 (0.36, 0.87) | 0.010 |
| Change in slope after week 13 |  |  |
| 5-year average | 1.02 (0.99, 1.05) | 0.240 |
| 2020 | 1.04 (1.00, 1.08) | 0.057 |
| 2020 relative to 5-year average | 1.02 (0.97, 1.08) | 0.424 |

## Framework for Enhanced Reporting of Interrupted Time Series (FERITS) *

| **Item** |  |  | **Page/location** |
| --- | --- | --- | --- |
| Title and Abstract |  | Study design (interrupted time series) indicated in the title or the abstract | 2 |
|  |  | Structured abstract recommended | 2 |
|  |  | Information on target population or study sample | 2 |
| Introduction | Background | Scientific background and explanation of rationale | 4 |
|  | Objectives | Specific objectives and hypotheses | 5 |
| Methods | Study Population | Eligibility criteria for participants, including criteria at different levels in recruitment/sampling plan (e.g., cities, clinics, subjects) | 5 |
|  |  | Methods of study population selection (such as codes or algorithms used to identify subjects from routine datasets) | 5 |
|  |  | Settings and locations where the data were collected | 5 |
|  | Study time-period | Start and end dates of the data included in the study, including reasons for selecting this date range and whether this was the full dataset available or if data was restricted. (Presentation of the full time series as a web appendix recommended) | 6 |
|  |  | Time intervals used (e.g. daily, monthly, annual) and the reason for selecting this interval | 6 |
|  |  | Clear definition of the preintervention period, the intervention point (including any transition period) and the post-intervention period | 6 |
|  | Intervention | Details of the intervention(s) and how and when they were actually administered, specifically including: |  |
|  |  | Why: Description of the rationale, theory, or goal of the elements essential to the intervention | 4 |
|  |  | What: Description of what was done including details of any policy changes, procedures, activities and information provided to participants | 4 |
|  |  | Who: Who developed, implemented and/or provided the intervention | 4 |
|  |  | How: Description of the modes of delivery of the intervention and whether it was provided individually, in a group or to a whole population | 4 |
|  |  | Where: Description of the type(s) of location(s) where the intervention occurred, including any necessary infrastructure or relevant features | 4 |
|  |  | When: Description of when the intervention was first announced, marketed and delivered, the number of times it was delivered and the duration. Did all groups receive the intervention at the same time | 4 |
|  |  | Tailoring and modifications: Description of any adaptations or modifications to the intervention during the course of the study | NA |
|  |  | Adherence: Report on whether the intervention was compulsory, whether adherence was assessed and any activities to increase compliance or adherence | NA |
|  | Outcomes | Clearly defined outcome measures | 5 |
|  |  | Data source(s) | 5 |
|  |  | Methods used to collect, process, record and extract data; any changes in data collection, processing or recording over time | 5 |
|  |  | Information on validity and reliability of outcome measures | 6 |
|  |  | Information on data quality, coverage and completeness over the duration of the study period; any changes in quality, coverage or completeness over time | 6 |
|  | History bias | Identification of co-interventions or other concurrent events that might affect the outcome; if no such events exist, clear statement that the intervention was independent of other changes | NA |
|  |  | Description of design adaptations to mitigate the risk of history bias e.g: adding a control series, using multiple phases or a using a multiple baseline design | 6 |
|  | Unit of Analysis | Description of the smallest unit that is being analysed to assess intervention effects (e.g., individual, group, or community) | 6 |
|  | Statistical Methods | Statistical methods used (e.g. segmented Poisson regression, ARIMA etc) | 6 |
|  |  | Appropriateness of a linear model, including description of any tests for linearity and any non-linear terms included | 7  We did not fit any non-linear terms in the model as the periods were relatively short. |
|  |  | Detailed description of the *a priori* impact model and why this was chosen, including allowance for: step or slope change effects, lagged effects, transition phase, floor or ceiling effects. Describe why this model is appropriate for the intervention and outcome under study | 6 |
|  |  | Adjustments for time varying confounders (including seasonality) | 6 |
|  |  | Assessment of autocorrelation and how this was handled | 6 |
|  |  | Description of any stratified or subgroup analyses | NA |
|  |  | Explanation of how missing data were addressed | 7 |
|  |  | Discussion of uncertainty in the primary statistical model and description of any additional sensitivity analyses | Table A, Table B |
|  |  | Statistical software or programs used | 6 |
| Results | Numbers analysed | Report on the number of participants (denominator) included in each analysis for each study condition throughout the study period, particularly when the denominators change for different outcomes | 5 |
|  | Population characteristics | Description of the baseline demographic and clinical characteristics of the intervention group and any control groups at baseline and throughout the study period (a table is recommended) | NA |
|  |  | Report on study group equivalence at baseline and statistical methods used to control for baseline differences | NA |
|  |  | Identification of differential changes in population characteristics between study groups throughout the study period and description of statistical methods used to control for differential changes | NA |
|  | Outcomes and estimation | For each primary and secondary outcome, a summary of results for each estimated, study condition, and the estimated effect size and a confidence interval to indicate the precision | Table 1, Table 2 |
|  |  | Report on both relative and absolute changes in the study outcomes following the intervention |  |
|  |  | Inclusion of null and negative findings | Table 2 |
|  |  | Inclusion of results from testing pre-specified causal pathways through which the intervention was intended to operate, if any | NA |
|  |  | Graphical presentation of the time series for each outcome with the regression line, pre-intervention time period, intervention points and post-intervention period clearly indicated | Figure 1, Figure 2 |
|  | Ancillary analyses | Summary of other analyses performed, including subgroup or restricted analyses and sensitivity analyses, indicating which are pre-specified or exploratory | NA |
|  | Adverse events | Summary of all important adverse events or unintended effects in each study condition (including summary measures, effect size estimates, and confidence intervals) | NA |
| Discussion | Interpretation | Interpretation of the results, taking into account study hypotheses, sources of potential bias, imprecision of measures, multiplicative analyses, and other limitations or weaknesses of the study | 14 |
|  |  | Discussion of results taking into account the mechanism by which the intervention was intended to work (causal pathways) or alternative mechanisms or explanations | 14 |
|  |  | Discussion of the success of and barriers to implementing the intervention, fidelity of implementation | NA |
|  |  | Discussion of research, programmatic, or policy implications | 17 |
|  | Generalizability | Generalizability (external validity) of the findings, taking into account the study population, the characteristics of the intervention, length of follow-up, incentives, compliance rates, specific sites/settings involved in the study, and other contextual issues | NA |
|  | Overall Evidence | General interpretation of the results in the context of current evidence and current theory | 14 |

* Lopez-Bernal J. Framework for Enhanced Reporting of Interrupted Time Series (FERITS) 2018. URL: http://www.equator-network.org/library/reporting-guidelines-under-development/reporting-guidelines-under-development-for-observational-studies/#92

## The STROBE and RECORD statements **

|  | **Item No.** | **STROBE items** | **Location in manuscript where items are reported** | **RECORD items** | **Location in manuscript where items are reported** |
| --- | --- | --- | --- | --- | --- |
| **Title and abstract** | | | | | |
|  | 1 | (a) Indicate the study’s design with a commonly used term in the title or the abstract (b) Provide in the abstract an informative and balanced summary of what was done and what was found | 2 | RECORD 1.1: The type of data used should be specified in the title or abstract. When possible, the name of the databases used should be included. | 2 |
|  |  |  |  | RECORD 1.2: If applicable, the geographic region and timeframe within which the study took place should be reported in the title or abstract. | 2 |
|  |  |  |  | RECORD 1.3: If linkage between databases was conducted for the study, this should be clearly stated in the title or abstract. | NA |
| **Introduction** | | | | | |
| Background rationale | 2 | Explain the scientific background and rationale for the investigation being reported | 4 |  |  |
| Objectives | 3 | State specific objectives, including any prespecified hypotheses | 5 |  |  |
| **Methods** | | | | | |
| Study Design | 4 | Present key elements of study design early in the paper | 6 |  |  |
| Setting | 5 | Describe the setting, locations, and relevant dates, including periods of recruitment, exposure, follow-up, and data collection | 6 |  |  |
| Participants | 6 | *(a) Cohort study* - Give the eligibility criteria, and the sources and methods of selection of participants. Describe methods of follow-up | NA | RECORD 6.1: The methods of study population selection (such as codes or algorithms used to identify subjects) should be listed in detail. If this is not possible, an explanation should be provided. | 5 |
|  |  | *Case-control study* - Give the eligibility criteria, and the sources and methods of case ascertainment and control selection. Give the rationale for the choice of cases and controls | NA |  |  |
|  |  |  |  | RECORD 6.2: Any validation studies of the codes or algorithms used to select the population should be referenced. If validation was conducted for this study and not published elsewhere, detailed methods and results should be provided. | NA |
|  |  | *Cross-sectional study* - Give the eligibility criteria, and the sources and methods of selection of participants | NA |  |  |
|  |  | *(b) Cohort study* - For matched studies, give matching criteria and number of exposed and unexposed | NA | RECORD 6.3: If the study involved linkage of databases, consider use of a flow diagram or other graphical display to demonstrate the data linkage process, including the number of individuals with linked data at each stage. | 7 |
|  |  | *Case-control study* - For matched studies, give matching criteria and the number of controls per case | NA |  |  |
| Variables | 7 | Clearly define all outcomes, exposures, predictors, potential confounders, and effect modifiers. Give diagnostic criteria, if applicable. | 5 | RECORD 7.1: A complete list of codes and algorithms used to classify exposures, outcomes, confounders, and effect modifiers should be provided. If these cannot be reported, an explanation should be provided. | 5 |
| Data sources/ measurement | 8 | For each variable of interest, give sources of data and details of methods of assessment (measurement).  Describe comparability of assessment methods if there is more than one group | 5 |  |  |
| Bias | 9 | Describe any efforts to address potential sources of bias | NA |  |  |
| Study size | 10 | Explain how the study size was arrived at | NA |  |  |
| Quantitative variables | 11 | Explain how quantitative variables were handled in the analyses. If applicable, describe which groupings were chosen, and why | 6 |  |  |
| Statistical methods | 12 | (a) Describe all statistical methods, including those used to control for confounding | 6 |  |  |
|  |  | (b) Describe any methods used to examine subgroups and interactions | 6 |  |  |
|  |  | (c) Explain how missing data were addressed | NA |  |  |
|  |  | (d) *Cohort study* - If applicable, explain how loss to follow-up was addressed | NA |  |  |
|  |  | *Case-control study* - If applicable, explain how matching of cases and controls was addressed | NA |  |  |
|  |  | *Cross-sectional study* - If applicable, describe analytical methods taking account of sampling strategy | NA |  |  |
|  |  | (e) Describe any sensitivity analyses | 7 |  |  |
| Data access and cleaning methods |  |  |  | RECORD 12.1: Authors should describe the extent to which the investigators had access to the database population used to create the study population. | 19 |
|  |  |  |  | RECORD 12.2: Authors should provide information on the data cleaning methods used in the study. | 7 |
| Linkage |  |  |  | RECORD 12.3: State whether the study included person-level, institutional-level, or other data linkage across two or more databases. The methods of linkage and methods of linkage quality evaluation should be provided. | 5 |
| **Results** | | | | | |
| Participants | 13 | (a) Report the numbers of individuals at each stage of the study (*e.g.*, numbers potentially eligible, examined for eligibility, confirmed eligible, included in the study, completing follow-up, and analysed)  (b) Give reasons for non-participation at each stage.  (c) Consider use of a flow diagram | NA | RECORD 13.1: Describe in detail the selection of the persons included in the study (*i.e.,* study population selection) including filtering based on data quality, data availability and linkage. The selection of included persons can be described in the text and/or by means of the study flow diagram. | 5 |
| Descriptive data | 14 | (a) Give characteristics of study participants (*e.g.*, demographic, clinical, social) and information on exposures and potential confounders  (b) Indicate the number of participants with missing data for each variable of interest  (c) *Cohort study* - summarise follow-up time (*e.g.*, average and total amount) | NA |  |  |
| Outcome data | 15 | *Cohort study* - Report numbers of outcome events or summary measures over time  *Case-control study* - Report numbers in each exposure category, or summary measures of exposure  *Cross-sectional study* - Report numbers of outcome events or summary measures | NA |  |  |
| Main results | 16 | (a) Give unadjusted estimates and, if applicable, confounder-adjusted estimates and their precision (e.g., 95% confidence interval). Make clear which confounders were adjusted for and why they were included | Table 1, Table 2, Table 3, Table 4 |  |  |
|  |  | (b) Report category boundaries when continuous variables were categorized | 6 |  |  |
|  |  | (c) If relevant, consider translating estimates of relative risk into absolute risk for a meaningful time period | NA |  |  |
| Other analyses | 17 | Report other analyses done—e.g., analyses of subgroups and interactions, and sensitivity analyses | 33 |  |  |
| **Discussion** | | | | | |
| Key results | 18 | Summarise key results with reference to study objectives | 14 |  |  |
| Limitations | 19 | Discuss limitations of the study, taking into account sources of potential bias or imprecision. Discuss both direction and magnitude of any potential bias | 15 | RECORD 19.1: Discuss the implications of using data that were not created or collected to answer the specific research question(s). Include discussion of misclassification bias, unmeasured confounding, missing data, and changing eligibility over time, as they pertain to the study being reported. | 15 |
| Interpretation | 20 | Give a cautious overall interpretation of results considering objectives, limitations, multiplicity of analyses, results from similar studies, and other relevant evidence | 14 |  |  |
| Generalisability | 21 | Discuss the generalisability (external validity) of the study results | NA |  |  |
| **Other Information** | | | | | |
| Funding | 22 | Give the source of funding and the role of the funders for the present study and, if applicable, for the original study on which the present article is based | 21 |  |  |
| Accessibility of protocol, raw data, and programming code |  | .. |  | RECORD 22.1: Authors should provide information on how to access any supplemental information such as the study protocol, raw data, or programming code. | 19 |

**Reference: Benchimol, Eric I., Liam Smeeth, Astrid Guttmann, Katie Harron, David Moher, Irene Petersen, Henrik T. Sørensen, Erik von Elm, Sinéad M. Langan, and RECORD Working Committee. "The REporting of studies Conducted using Observational Routinely-collected health Data (RECORD) statement." PLoS Med 12, no. 10 (2015): e1001885.

*Checklist is protected under Creative Commons Attribution (CC BY) license.
